# Retraction: TNF-α Induces Cytosolic Phospholipase A_2_ Expression in Human Lung Epithelial Cells via JNK1/2- and p38 MAPK-Dependent AP-1 Activation

**DOI:** 10.1371/journal.pone.0268929

**Published:** 2022-05-19

**Authors:** The PLOS ONE Editors

Following the publication of this article [1] and correction [2], additional concerns were raised regarding results reported in multiple figures. Specifically, the following results appear similar despite representing different experimental conditions:

- The lower cPLA_2_ panel in Fig 2A and the cPLA_2_ panel in Fig 4A.
- The p38 panel in Fig 4C and the ATF2 panel in Fig 7A with colour levels adjusted.
- Lanes 2–4 of the GAPDH for cPLA_2_ panel in Fig 4C, the GAPDH panel for c-Jun in Fig 6C, and lanes 3–5 of the GAPDH panel for cPLA_2_ (upper set) in Fig 6C.
- Lanes 4–6 of the GAPDH panel for cPLA_2_ in Fig 4C and the GAPDH panel for c-Fos in Fig 6C.
- The c-Jun and p-ATF2 panels in Fig 7B with colour levels adjusted.

The corresponding author stated that multiple western blot experiments were performed and imaged at the same time. They acknowledged that errors occurred in the preparation of the figures and annotation of raw images, and apologized for these errors. The corresponding author accepted that the issues with the published figures cannot be fully resolved, and requested that the article be retracted.

In light of the concerns affecting multiple figure panels and the errors in the raw underlying data that question the reliability of these data, the *PLOS ONE* Editors retract this article.

ITL, CCL, LDH and CMY agreed with the retraction. SEC and YCH either did not respond directly or could not be reached.
